# Cone Beam Computed Tomography Evaluation of the Diagnosis, Treatment Planning, and Long-Term Followup of Large Periapical Lesions Treated by Endodontic Surgery: Two Case Reports

**DOI:** 10.1155/2013/564392

**Published:** 2013-05-22

**Authors:** Vijay Shekhar, K. Shashikala

**Affiliations:** Department of Conservative Dentistry and Endodontics, D.A.P.M. R.V. Dental College and Hospital, Bangalore, Karnataka 560078, India

## Abstract

The aim of this case report is to present two cases where cone beam computed tomography (CBCT) was used for the diagnosis, treatment planning, and followup of large periapical lesions in relation to maxillary anterior teeth treated by endodontic surgery. Periapical disease may be detected sooner using CBCT, and their true size, extent, nature, and position can be assessed. It allows clinician to select the most relevant views of the area of interest resulting in improved detection of periapical lesions. CBCT scan may provide a better, more accurate, and faster method to differentially diagnose a solid (granuloma) from a fluid-filled lesion or cavity (cyst). In the present case report, endodontic treatment was performed for both the cases followed by endodontic surgery. Biopsy was done to establish the confirmatory histopathological diagnosis of the periapical lesions. Long-term assessment of the periapical healing following surgery was done in all the three dimensions using CBCT and was found to be more accurate than IOPA radiography. It was concluded that CBCT was a useful modality in making the diagnosis and treatment plan and assessing the outcome of endodontic surgery for large periapical lesions.

## 1. Introduction

The most common pathologic conditions that involve teeth are the periapical lesions, usually composed of solid soft tissue (granulomas) or semisolid, liquefied cystic area (cyst). They are the result of a localized inflammatory reaction to infection within the root canal system reducing mineral density of the affected periapical bone, resulting in resorption identified as radiolucencies in radiographs. It is essential to correctly diagnose these lesions as the choice of treatment is dependent on it [1]. Root canal therapy is accepted as the most effective conservative method for treating periapical lesions [2]. Periapical granuloma may heal without surgical treatment if given the opportunity; however, sometimes it may require surgical removal. It is controversial whether cysts heal with nonsurgical treatment because until a biopsy is taken, the clinician does not know the histologic diagnosis. Once a biopsy is taken, the treatment is no longer only nonsurgical. Periapical cyst usually requires surgical removal of the cyst.

The clinical, radiographic, and histological diagnosis of periapical lesions has been a challenge, and differentiating between the various periapical lesions remains an open research problem. Current diagnostic methods help in fair assessment of accurate size and nature of a periapical lesion which determine the treatment and prognosis of the tooth in question. Although, invasive post hoc biopsy is the only reliable method of diagnosis currently available, clinicians are aware of the difficulty in obtaining biopsies in routine clinical practice. Attempt to diagnose, the lesion before surgery with intraoral periapical (IOPA) radiographs, contrast media, Papanicolaou smears, and albumin tests have proven to be inaccurate. Thus, there is a need for a noninvasive method to diagnose lesions involving the periapical area. Recently with the advent of imaging modalities such as digital radiography, densitometry methods, computed tomography (CT), magnetic resonance imaging (MRI), ultrasound, and cone beam computed tomography (CBCT), differences in density may permit more accurate preoperative diagnosis [1, 3, 4].

The therapeutic protocol to treat diseases of endodontic origin is based on the evaluation of pathologic and clinical characteristics frequently complemented by radiographic findings using high-quality diagnostic radiographs. At present, IOPA radiography is the technique of choice for diagnosing, managing, and assessing endodontic diseases like periapical lesions. Radiographs provide bidimensional images of three-dimensional (3D) structures that do not permit the evaluation of bone thickness, determination of the size, and localization of periapical lesions. As a result of superimposition, IOPA radiographs reveal limited aspects of the 3D anatomy. In addition, there may also be geometric distortion of the anatomical structures being imaged. Limitations of IOPA radiographs for detecting periapical lesions include that, to be visible radiographically, cortical plate must be engaged. If confined within cancellous bone, they are usually not detected. A lesion of certain size can be detected in region covered by thin cortex but cannot be detected in region covered by thicker cortex. Many subsequent studies since that time have underscored the difficulty of detecting periapical lesions using IOPA radiographs. Therefore, considering some limitations on conventional radiography for detection of periapical bone lesions, advanced imaging methods such as CBCT might add benefits to endodontics and offer a higher quality for the diagnosis, treatment planning, and prognosis [1, 2, 4–9].

CBCT is a dentomaxillofacial imaging technique, which potentially provides dentistry with a practical tool for noninvasive and 3D reconstruction of teeth and their surrounding structures. CBCT exposes an object to multiple cone-shaped beams to acquire volume of the object, and later serial section images are obtained in coronal, sagittal, and axial planes, making possible the 3D interpretation. Thus, the clinician can visualize morphologic features and pathologies from different 3D perspectives [2]. Periapical disease may be detected sooner using CBCT compared with periapical views, and the true size, extent, nature, and position of periapical lesions can be assessed. Its other applications include visualization of root canal anatomy, assessment of true nature of the alveolar bone topography around teeth, treatment planning for periapical surgery, and the assessment of treatment outcomes. CBCT scans are desirable to assess teeth prior to periapical surgery, as the thickness of the cortical and cancellous bone and inclination of roots in relation to the surrounding jaw can be accurately determined, and the relationship of vital anatomical structures nerve to the root apices may also be clearly visualized [5, 10].

CBCT systems used in endodontics provide small field of view images at low dose with rapid scan time and sufficient spatial resolution. Various studies have found CBCT to be clinically superior to IOPA radiography for the detection of periapical lesions [1, 4–6, 11]. Thus, CBCT scan may provide safer, faster, and more accurate method for differential diagnosis of the periapical lesions. This allows clinician to decide, based on the type of periapical lesion, whether surgery is necessary or not, without the invasive biopsy procedure and waiting to see if healing has occurred (recall period). Evaluation of a periapical lesion with CBCT changes the estimation of size and choice of treatment among endodontists compared to IOPA radiography. Selecting the most appropriate choice of treatment using the most accurate imaging modality would ultimately reduce cost and morbidity significantly in patients undergoing endodontic therapy, and the lesion may be managed more effectively. However, scientific consensus maintains that true nature of a periapical lesion is accurately identified by histopathology only, which is considered to be the “gold standard” for achieving final confirmatory diagnosis of periapical lesions [1, 2, 4, 12].

A thorough history, clinical examination, and good quality radiographs are essential for preoperative diagnosis of teeth scheduled to undergo apical surgery [6]. In the currently available studies, no clear indications for the use of CBCT scans in diagnosis before apical surgery have been identified, and not as much emphasis has been placed on the applications of CBCT to endodontics, such as in the diagnosis, treatment planning, management, and followup of large periapical lesions requiring endodontic surgery [10]. 

The treatment outcome should be determined at follow-up examinations for at least 4 years, when it is established whether the preexisting periapical radiolucency has completely disappeared. IOPA radiographs have been widely used for root canal treatment followup [2]. Recently, CBCT has found to be useful in measuring the bone density before and after endodontic treatment [13]. Thus, CBCT can be used for the assessment of periapical healing following root canal treatment and endodontic surgery. CBCT provides an effective and safe way of producing 3D information of individual teeth and adjacent structures and may in time change the way in which the outcome of endodontic treatment is assessed [5]. 

The objective of this case report is to present two cases where CBCT was used for the diagnosis, treatment planning, and long-term followup of healing of large periapical lesions in relation to maxillary anterior teeth, treated by endodontic surgery, compared to IOPA radiographs.

## 2. Case Reports

### 2.1. History and Diagnosis

#### 2.1.1. Case 1

**Figure 1:**
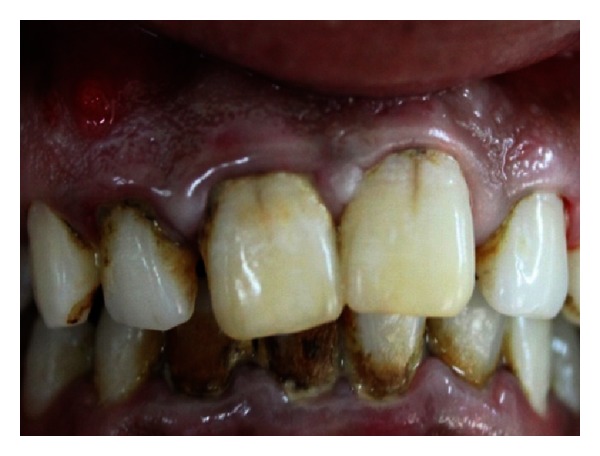
Preoperative photo showing presence of sinus tract with respect to 12 and 13.

**Figure 2:**
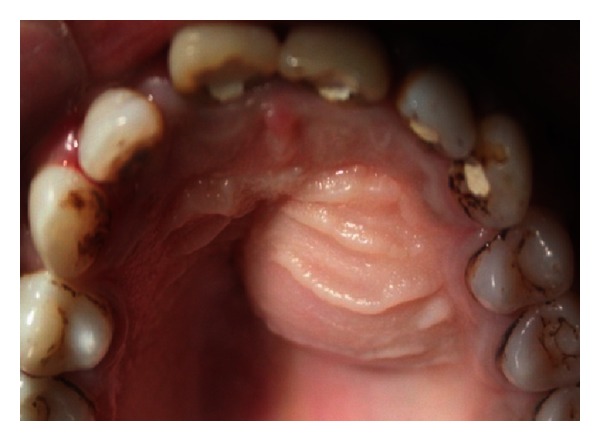
Pre-operative photo showing swelling of the palatal soft tissue.

A 27-year-old male patient reported with the chief complaint of swelling in the upper front right region of the mouth 1 month ago. He had a history of trauma to right upper anterior region 12 years back. The clinical examination revealed intraoral swelling in palatal region accompanied with salty discharge with respect to teeth 11, 12, and 21, and a sinus opening with respect to the apex on buccal aspect of 12 and 13 (Figures 1 and 2). The teeth were tender on percussion and nonvital with Ellis and Davey class 4 trauma. Palpation of right upper anterior region was painful. Teeth 11 and 12 had grade 1 mobility.

**Figure 3:**
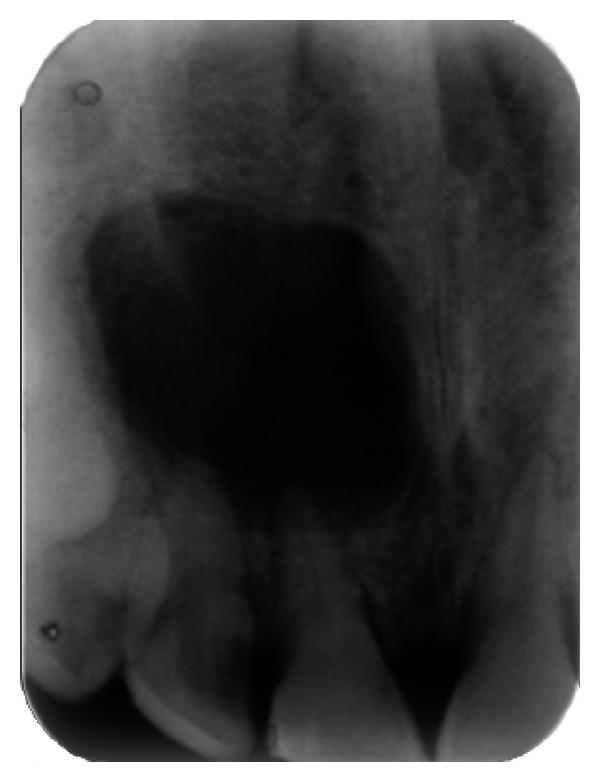
Pre-operative IOPA radiograph showing well-defined large periapical lesion.

**Figure 4:**
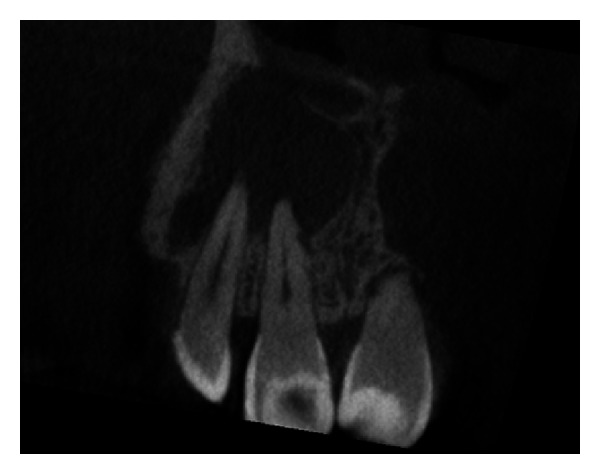
Pre-operative CBCT image in coronal plane showing presence of a large periapical lesion.

**Figure 5:**
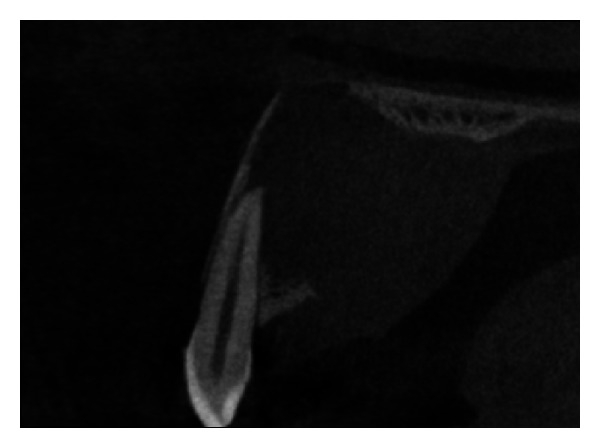
Pre-operative CBCT image in sagittal plane.

**Figure 6:**
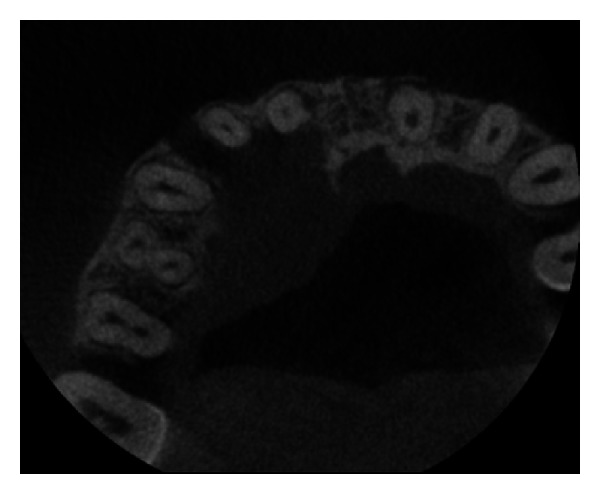
Pre-operative CBCT image in axial plane showing disruption of buccal and palatal cortex.

**Figure 7:**
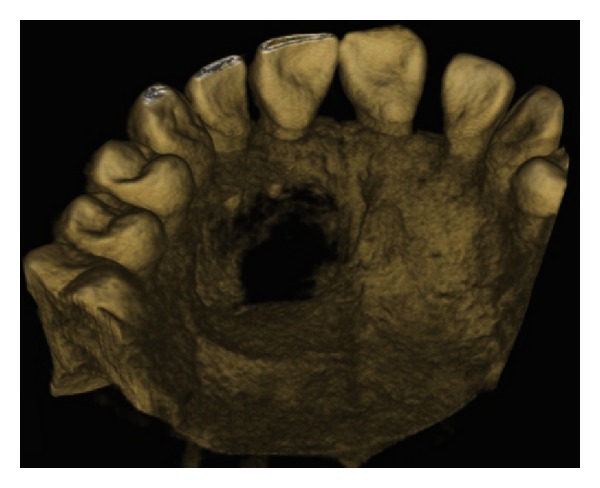
Reconstructed 3D CBCT image.

The IOPA radiographic examination revealed a large radiolucent lesion with a well-defined border in the periapical area of teeth 11, 12, and 21 suggestive of periapical cyst (Figure 3). CBCT examination revealed presence of periapical radiolucency with respect to 11, 12, 13, and 21 in sagittal, coronal, and axial planes and reconstructed three-dimensional image (Figures 4, 5, 6, and 7). Oral and maxillofacial radiologist categorized the CBCT images, based on the radiodensity as periapical granuloma.

#### 2.1.2. Case 2

A 22-year-old male patient reported with the chief complaint of pus discharge from upper right front region of mouth for 4-5 years. The clinical examination revealed discolored fractured crown fracture with respect to tooth 11 while tooth 12 was displaced (Figure 27). Teeth 11 and 12 were nonvital with Ellis and Davey class 4 trauma.

The IOPA radiographic examination revealed large radiolucent lesion with a well-defined sclerotic border in the periapical area of teeth 11 and 12, along with displacement of these teeth suggestive of periapical cyst (Figure 28). CBCT examination revealed presence of periapical radiolucency with respect to 11 and 12 in sagittal, coronal, and axial planes and reconstructed three-dimensional image (Figures 29, 30, 31, and 32). Oral and maxillofacial radiologist categorized the CBCT images, based on the radiodensity as periapical cyst. 

### 2.2. Management

**Figure 8:**
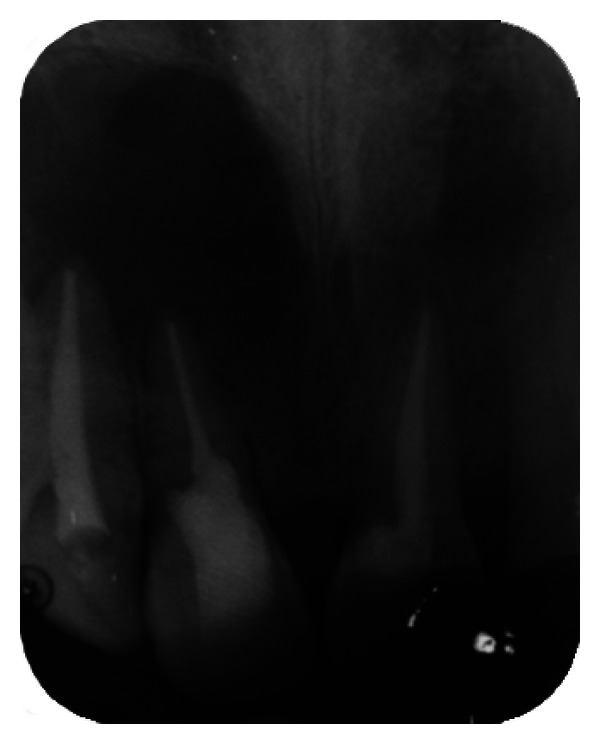
Postobturation IOPA radiograph.

**Figure 9:**
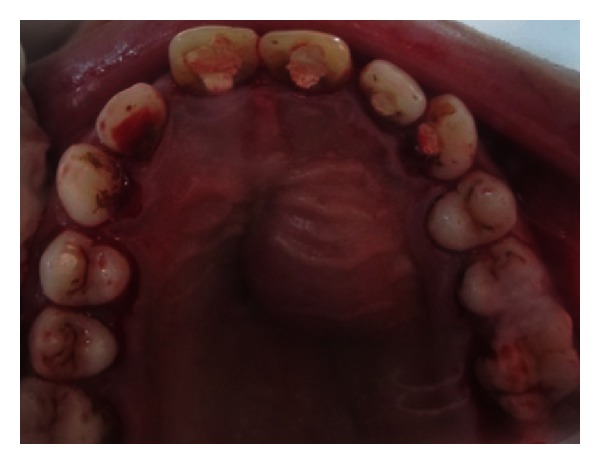
Surgical incision on palatal aspect.

**Figure 10:**
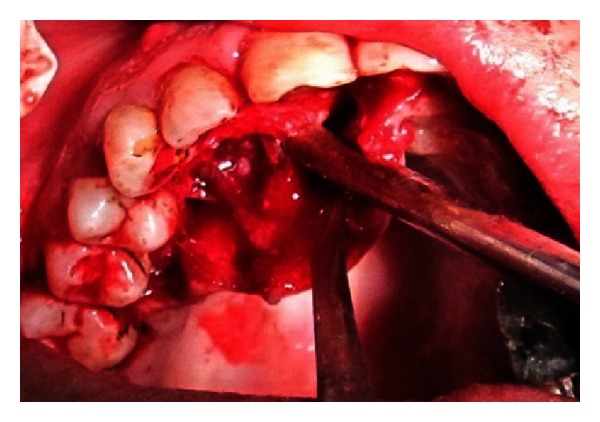
Flap reflection.

**Figure 11:**
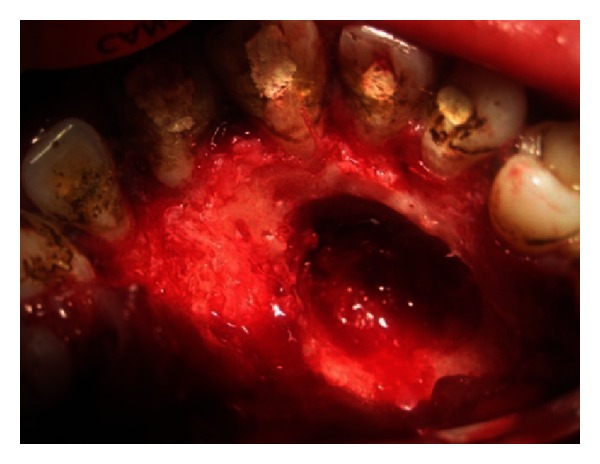
Flap retraction and exposure of bony defect.

**Figure 12:**
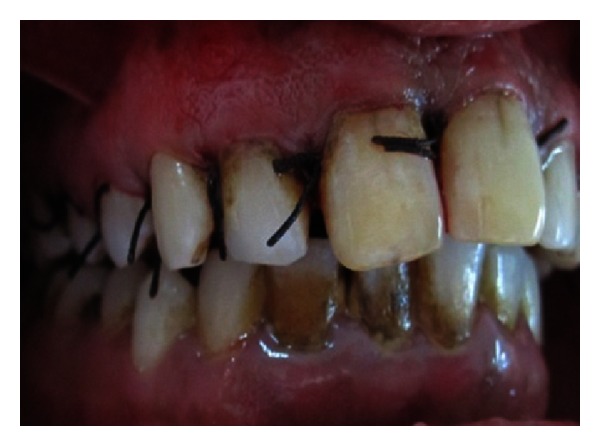
Suturing.

**Figure 13:**
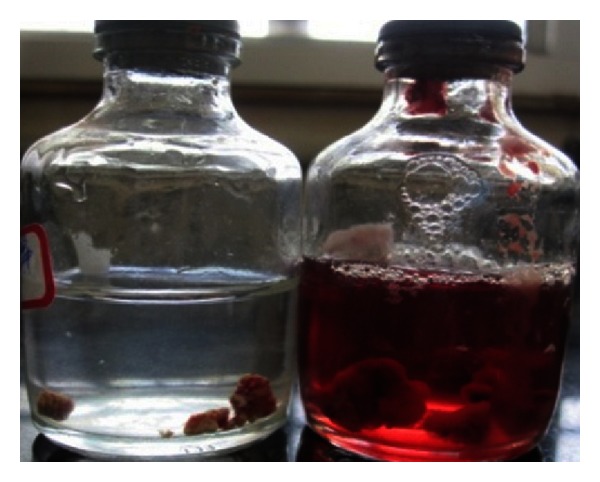
Lesional tissue stored in formalin for histopathological examination.

**Figure 14:**
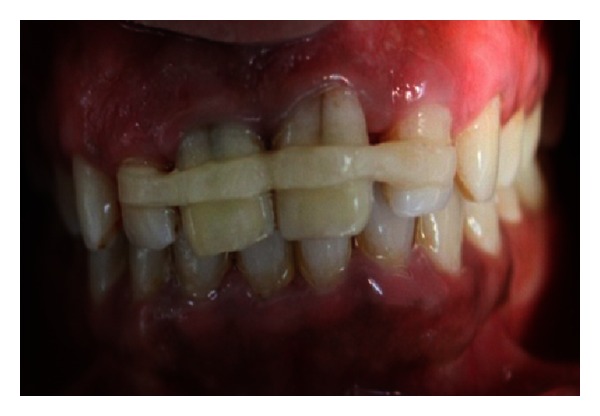
Splinting of mobile teeth using composite.

**Figure 15:**
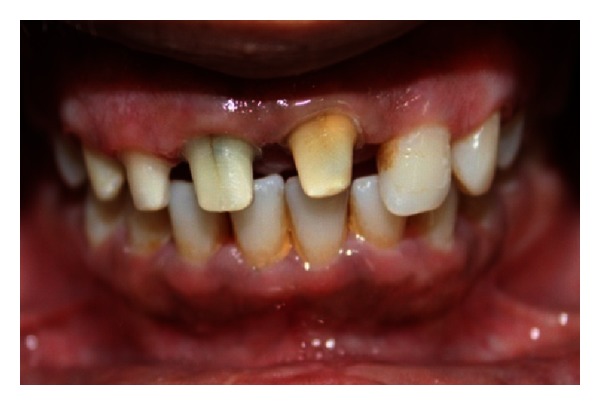
Tooth preparation.

**Figure 16:**
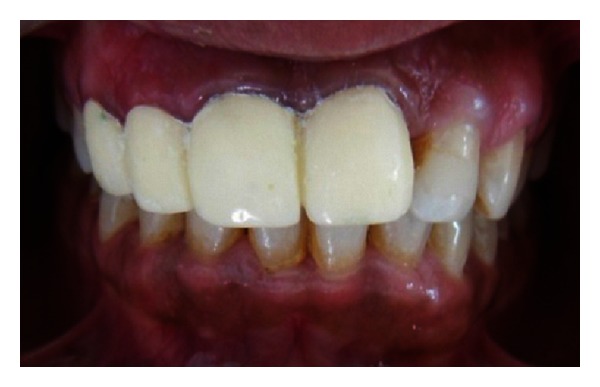
Temporization with acrylic crowns.

**Figure 17:**
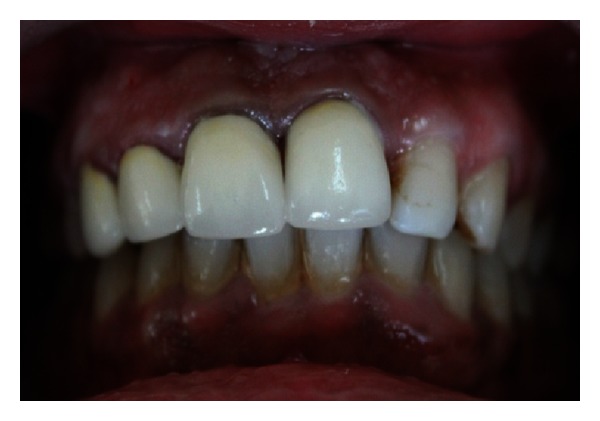
PFM crown cementation on 11, 12, 13, and 21.

The endodontic treatment was performed as per standard procedures in both cases under rubber dam isolation (Figures 8 and 33). All the canals were instrumented with hand K files and irrigated with 5.25% sodium hypochlorite. The root canals were obturated using gutta percha, and access cavity was sealed with temporary restorative material. This was followed by endodontic surgery (apicoectomy) (Figures 9, 10, 11, 12, 34, 35, 36, and 37), during which biopsy specimens were taken for histopathologic examination (Figures 13 and 38). Splinting with composite was done in the first case of the mobile teeth for 4 weeks (Figure 14). Then tooth preparation was done followed by temporisation of the involved teeth with acrylic crowns (Figures 15, 16, 39, and 40). Finally, porcelain fused to metal (PFM) crowns were cemented on the involved teeth (Figures 17 and 41). 

### 2.3. Histopathologic Examination

**Figure 18:**
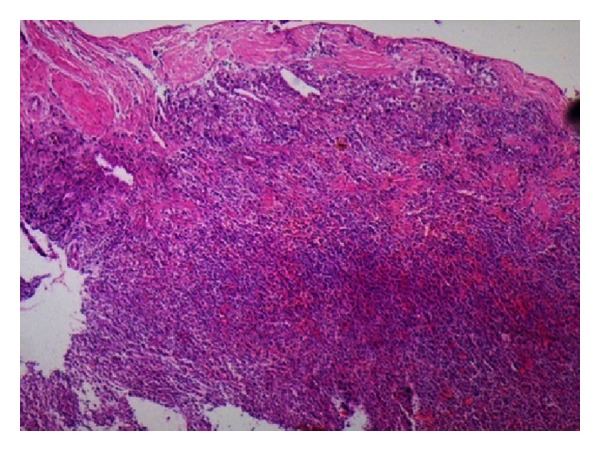
Histopathology report: periapical granuloma.

Histopathologic examination done in the first case revealed fibrous connective tissue which was inflamed, dense aggregate of chronic inflammatory cells, dilated and congested blood vessels and hemorrhagic foci, many Russell bodies, and few giant cells. No evidence of epithelium was present in the given sections. Oral pathologist diagnosed the specimen as periapical granuloma (Figure 18). Histopathologic examination done in the second case revealed cystic lining of nonkeratinized stratified squamous epithelium of varying thickness, an “arcading” pattern of proliferation, fibrovascular connective tissue wall which contained hemorrhagic areas and a dense inflammatory infiltrate of lymphocytes, plasma cells, and neutrophils. Oral pathologist diagnosed the specimen as infected periapical cyst (Figure 42).

### 2.4. Followup

**Figure 19:**
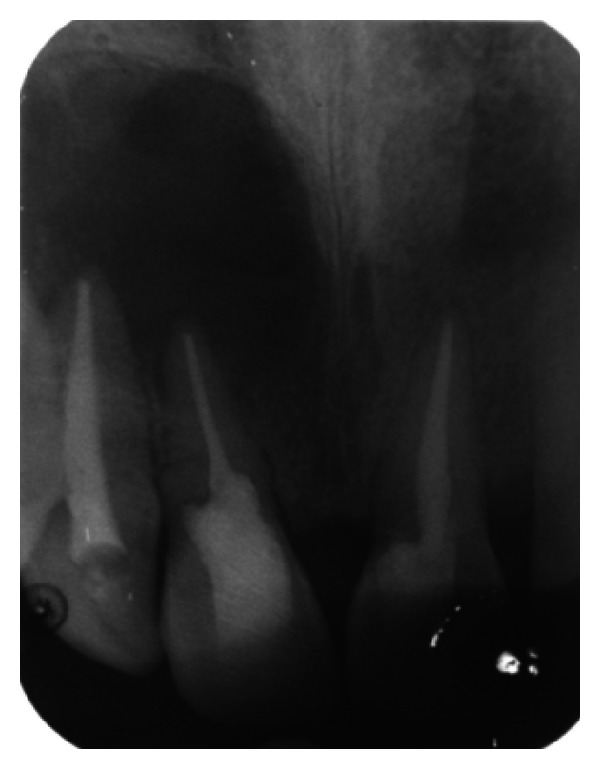
1-month followup IOPA radiograph.

**Figure 20:**
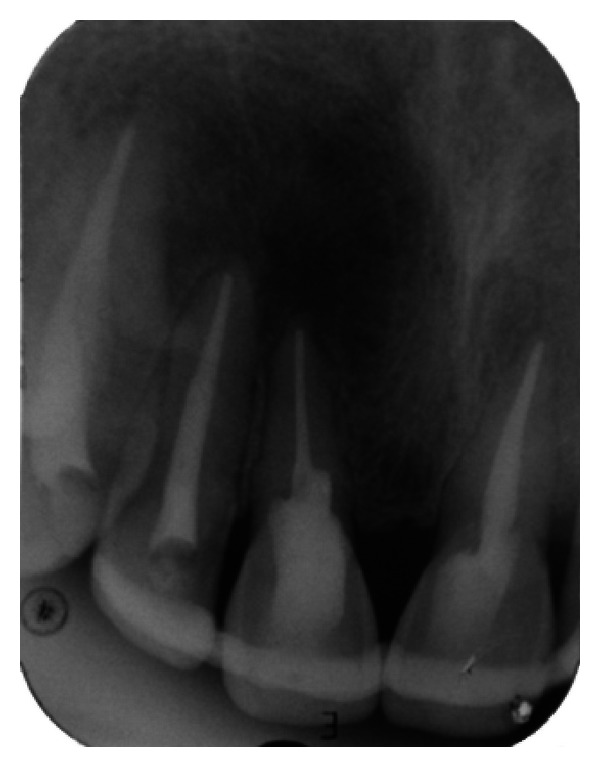
6-month followup IOPA radiograph.

**Figure 21:**
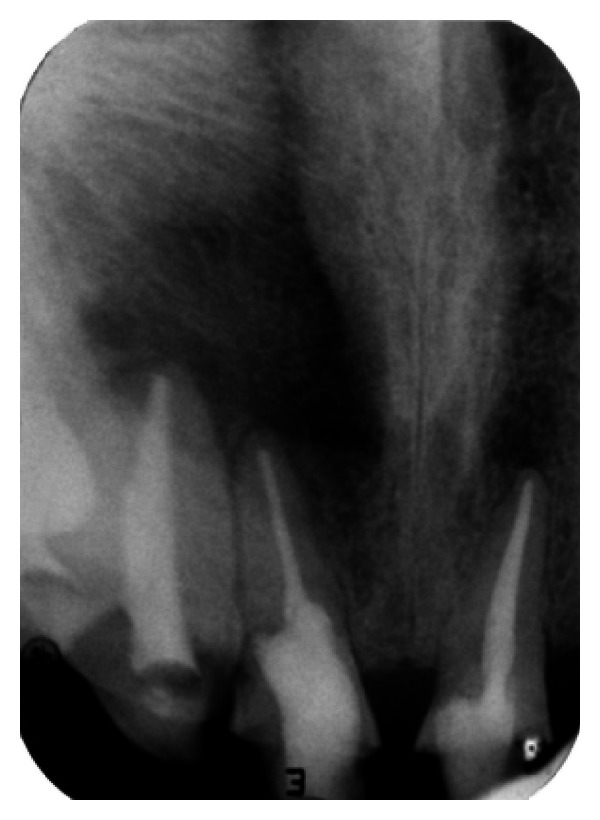
12-month followup IOPA radiograph.

**Figure 22:**
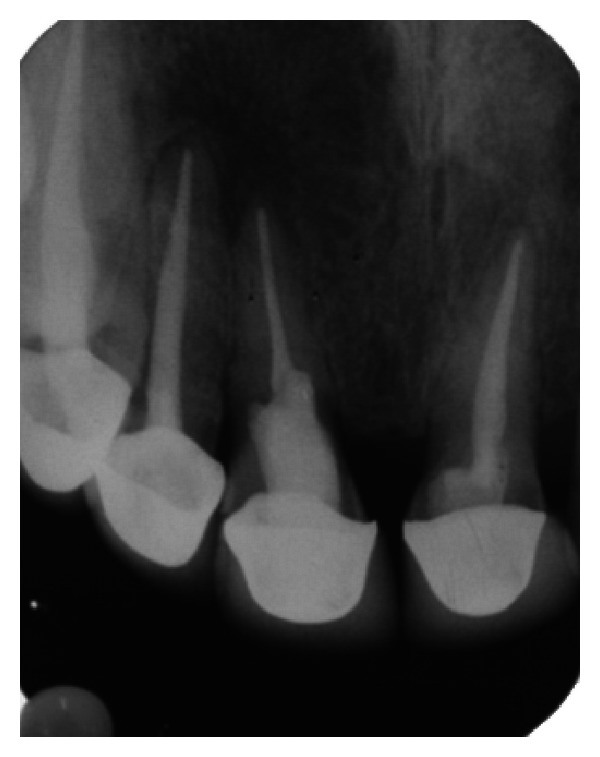
18-month followup IOPA radiograph.

**Figure 23:**
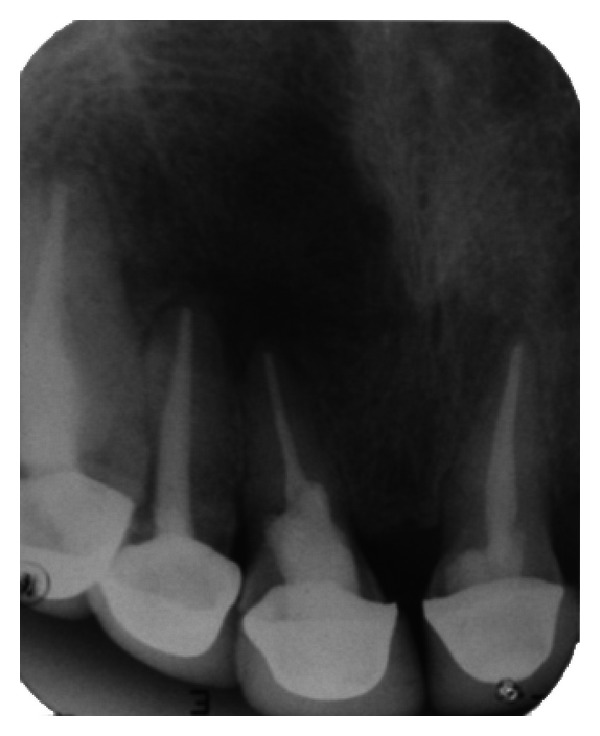
24-month followup IOPA radiograph.

**Figure 24:**
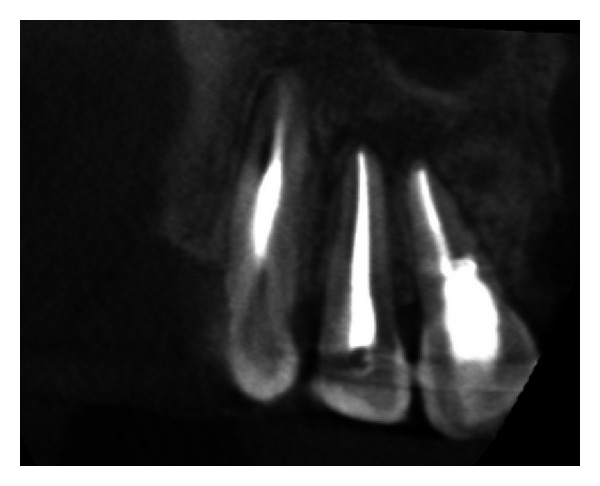
1-year followup CBCT image in coronal plane.

**Figure 25:**
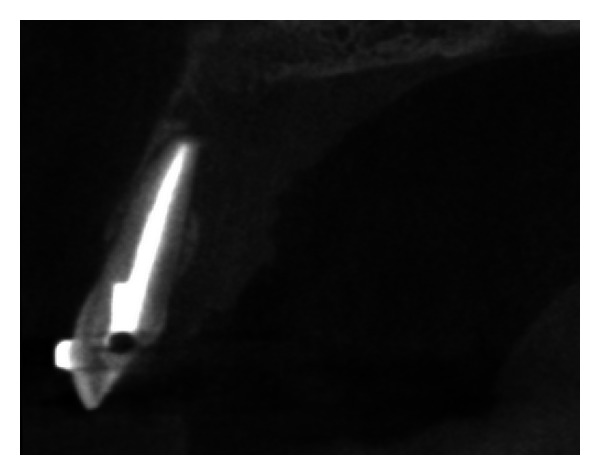
1-year followup CBCT image in sagittal plane.

**Figure 26:**
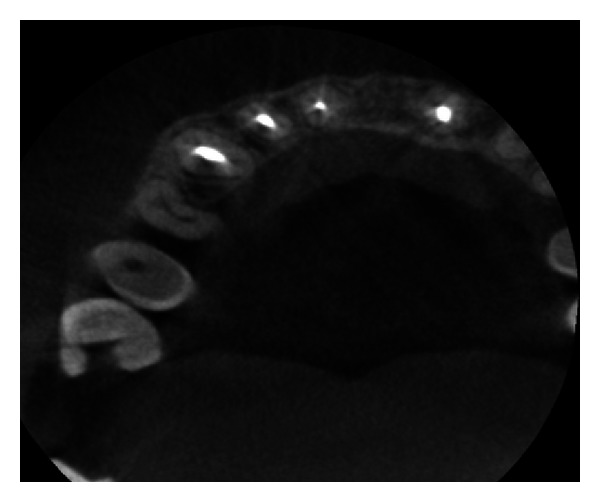
1-year followup CBCT image in axial plane showing establishment of buccal and palatal cortical plates.

**Figure 27:**
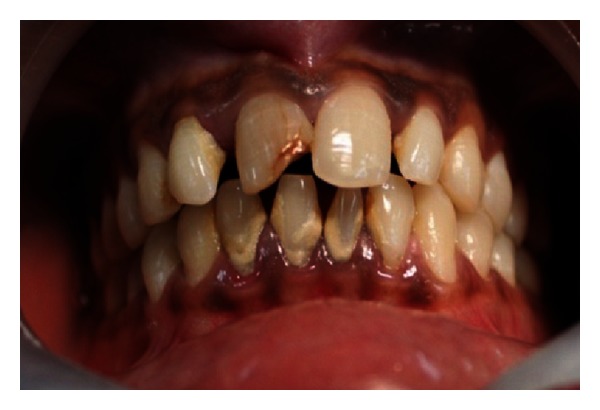
Pre-operative photo showing fractured and discolored 11 and displaced 12.

**Figure 28:**
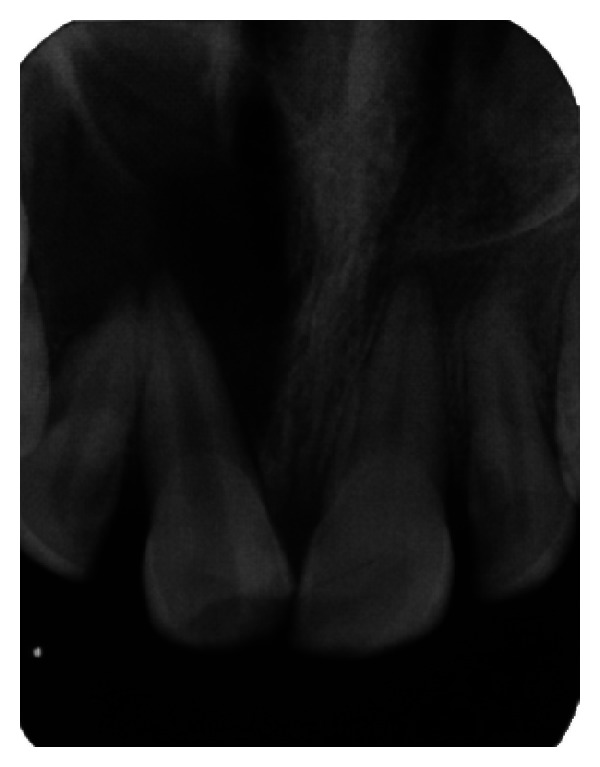
Pre-operative IOPA radiograph showing well-defined large periapical lesion.

**Figure 29:**
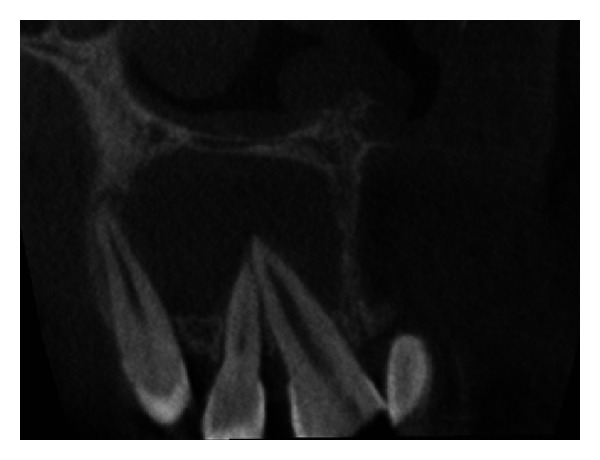
Pre-operative CBCT image in coronal plane.

**Figure 30:**
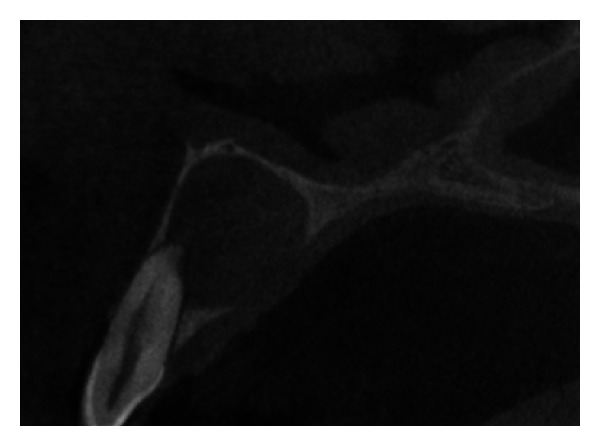
Pre-operative CBCT image in sagittal plane.

**Figure 31:**
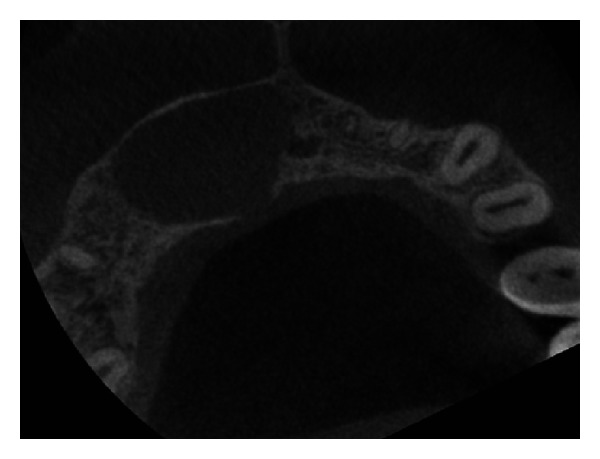
Pre-operative CBCT image in axial plane.

**Figure 32:**
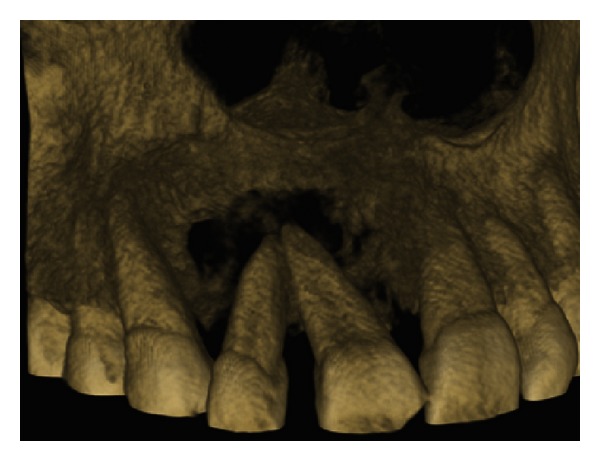
Reconstructed 3D CBCT image.

**Figure 33:**
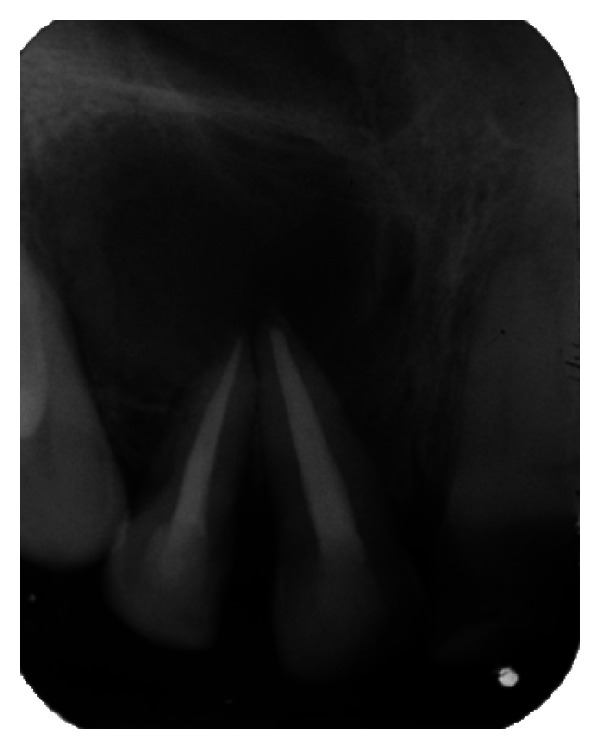
Postobturation IOPA radiograph.

**Figure 34:**
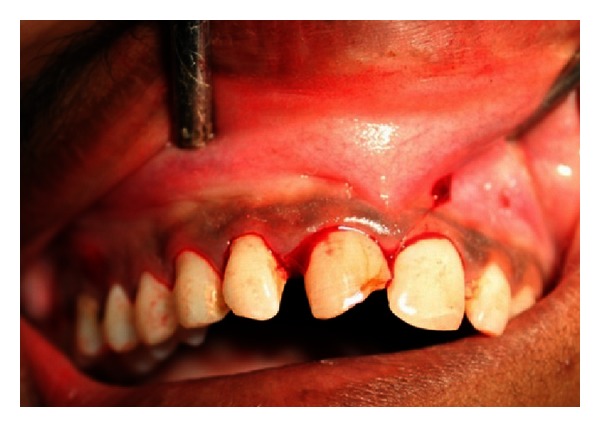
Surgical incision.

**Figure 35:**
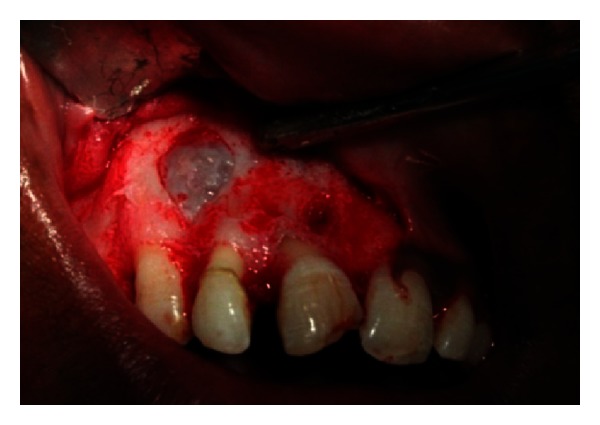
Flap retraction and exposure of the cyst.

**Figure 36:**
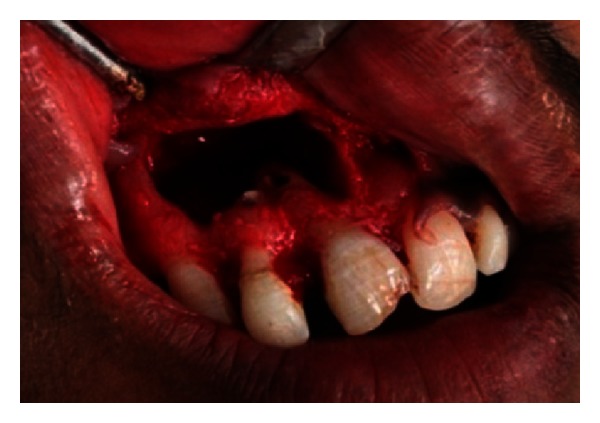
Apicoectomy.

**Figure 37:**
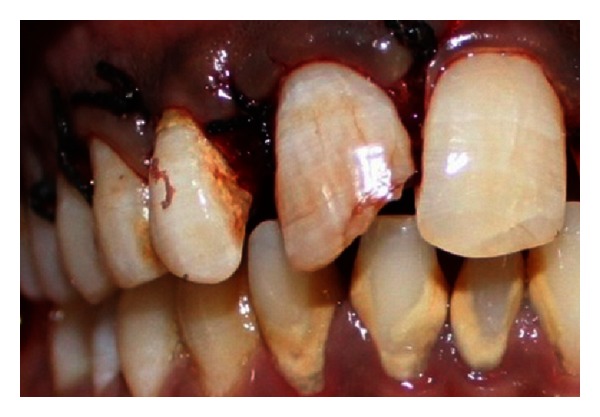
Suturing.

**Figure 38:**
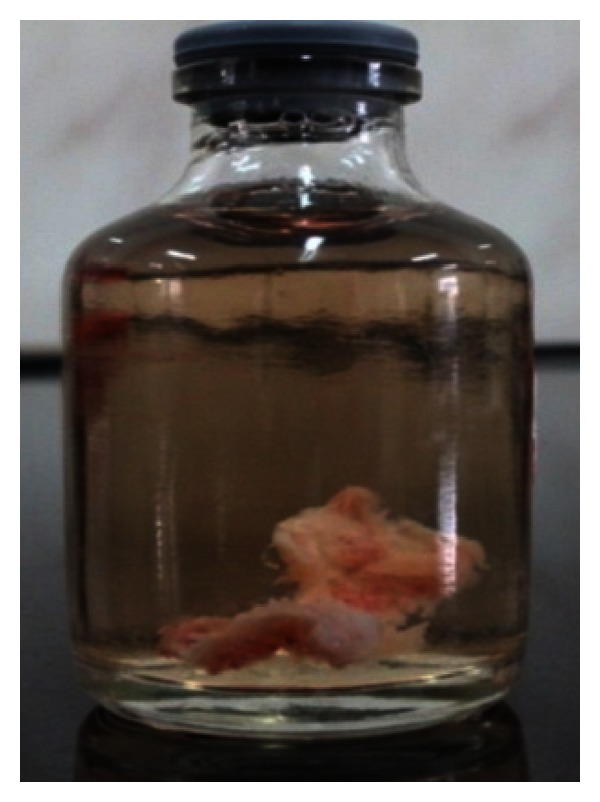
Lesional tissue stored in formalin.

**Figure 39:**
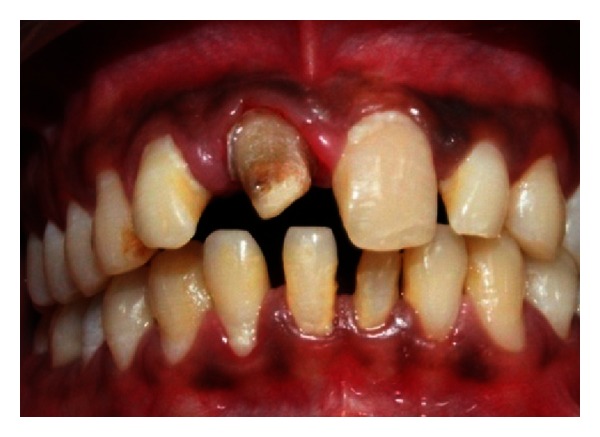
Tooth preparation.

**Figure 40:**
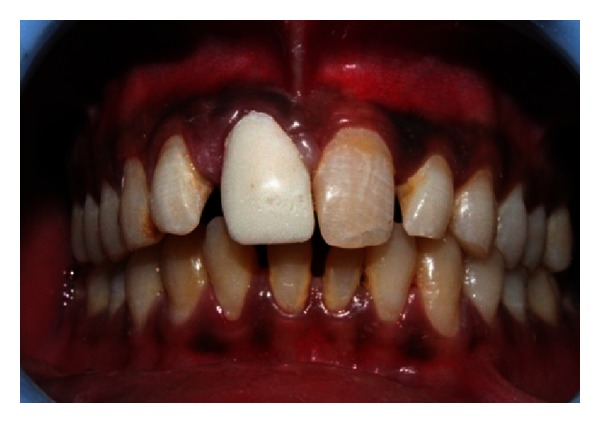
Temporisation.

**Figure 41:**
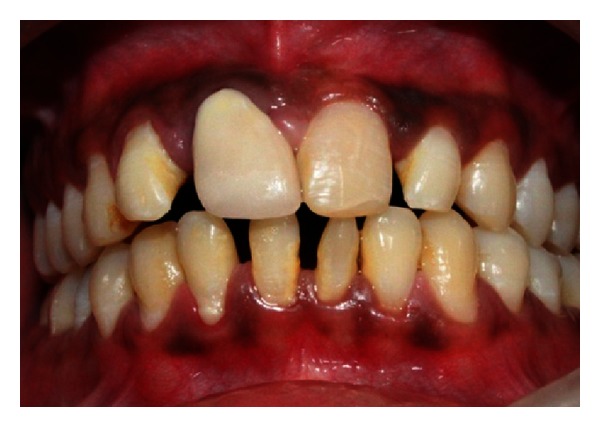
PFM crown cementation.

**Figure 42:**
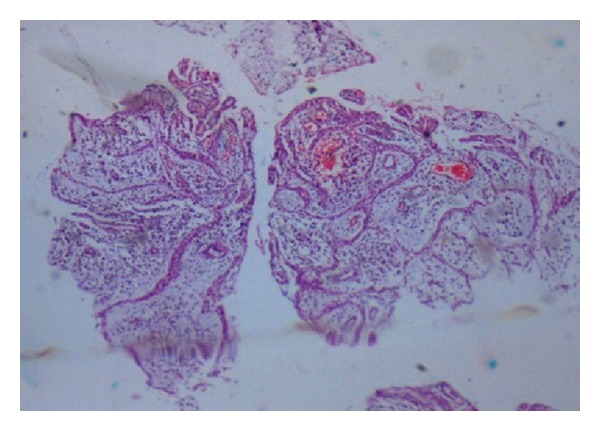
Histopathology report: radicular cyst.

**Figure 43:**
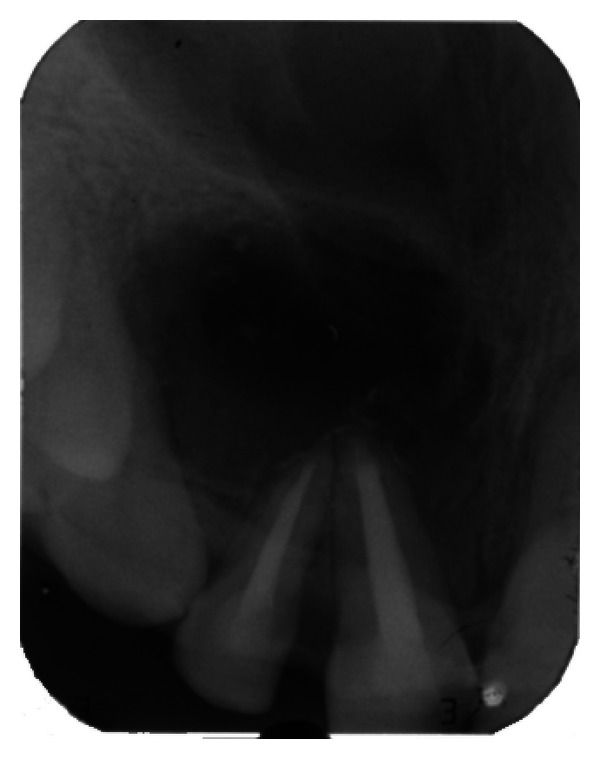
1-month followup IOPA radiograph.

**Figure 44:**
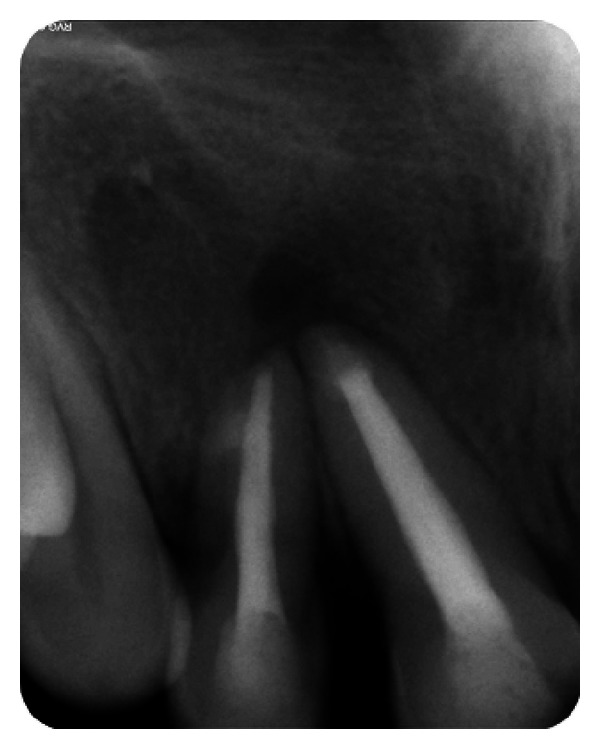
6-month followup IOPA radiograph.

**Figure 45:**
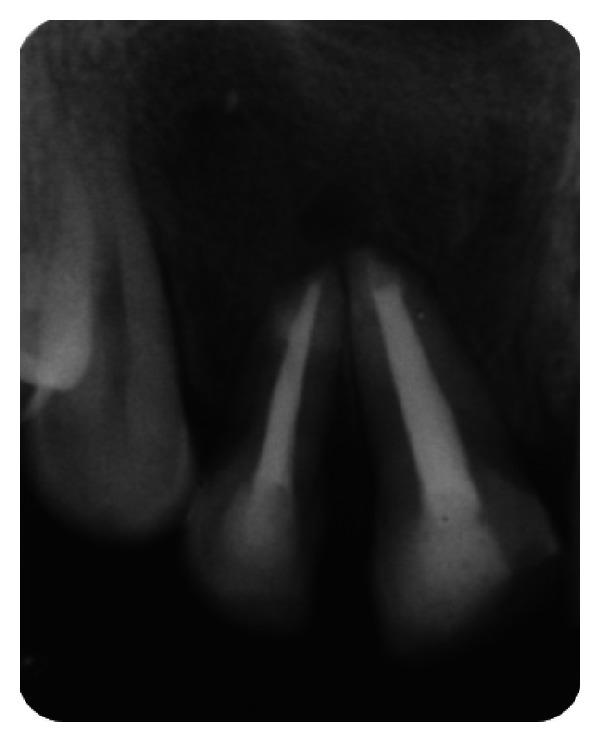
12-month follow-up IOPA radiograph.

**Figure 46:**
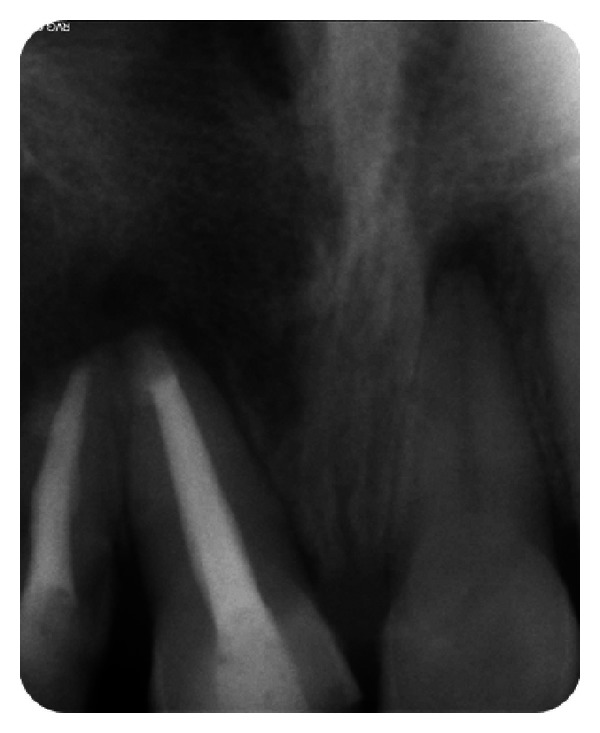
18-month followup IOPA radiograph.

**Figure 47:**
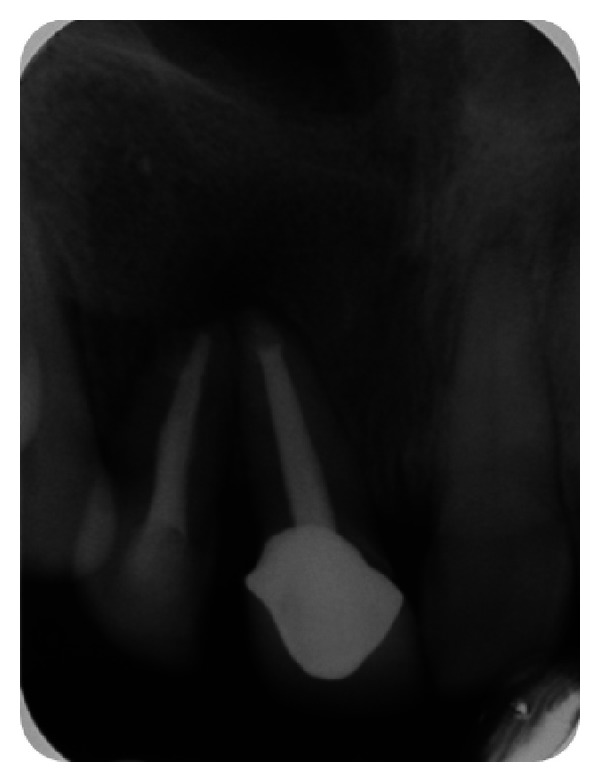
24-month followup IOPA radiograph.

**Figure 48:**
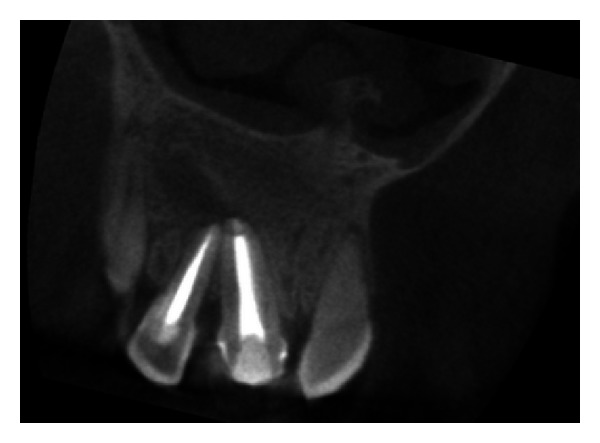
1-year followup CBCT image in coronal plane.

**Figure 49:**
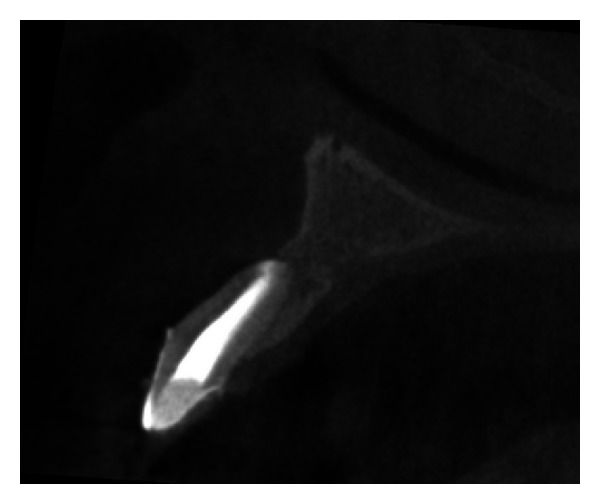
1-year followup CBCT image in sagittal plane.

**Figure 50:**
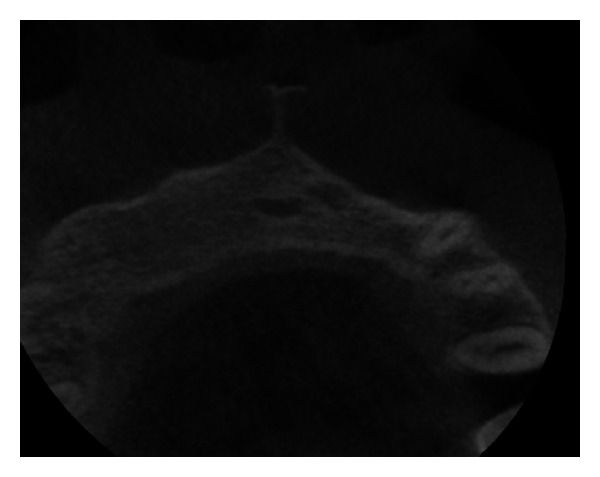
1-year followup CBCT image in axial plane showing establishment of the buccal and lingual cortical plates.

The patient was recalled regularly for two years for followup done by clinical and radiographic examination using IOPA radiographs and CBCT. The IOPA radiographs at one month, six months, 12 months, 18 months, and 24 months showed a decrease in the size of periapical radiolucency (Figures 19, 20, 21, 22, 23, 43, 44, 45, 46, and 47). In both cases, at the end of two years, it was noted that at the periapex of the involved teeth, there was a small radiolucency termed as the periapical scar. Healing with periapical scar is acceptable and common with large periapical lesions. The CBCT examination done at the end of one year revealed a remarkably reduced size of the periapical radiolucency in all the three planes: coronal, sagittal, and axial in both cases (Figures 24, 25, 26, 48, 49, and 50). Thus, both clinical and radiographic examination revealed a complete healing of the periapical lesions at the end of two years.

## 3. Discussion

This case report used three study tools for the diagnosis of two large periapical lesions: 3D imaging by CBCT (9500 Cone Beam 3D System), conventional two-dimensional imaging by IOPA radiography, and histopathological examination by biopsy. The initial diagnosis and treatment planning for management of periapical lesions were done using IOPA radiographs and gray scale value measurements with CBCT. For the management, endodontic treatment was performed for all cases followed by endodontic surgery. The radiographic findings were compared to surgical biopsy report which established the confirmatory histopathological diagnosis of the periapical lesions. Followup was done for the assessment of treatment outcome following surgery of the large periapical lesions, using CBCT and IOPA radiography.

A diagnostic test should exhibit validity and reliability if it is to be useful. Determining whether the periapical radiolucency is a cyst or granuloma cannot be done with IOPA radiographs alone. Until a biopsy is taken, the clinician does not know the confirmatory histopathologic diagnosis, but it is an invasive procedure requiring surgical intervention [1, 2, 4, 12]. Shortcomings in histological technique are that invasive surgery is mandatory to obtain specimen, lesion is curetted, often only small, multiple pieces are obtained, and false diagnosis is possible in lesions with epithelial lining that appears to be lining a lumen which is the histologic picture of bay/true cyst. To be accurate, serial sections through tooth with lesion attached are necessary. It is expensive, time consuming, and rarely done.

CBCT is a recent diagnostic modality whose software allows clinician to view reconstructed slices of data in 3D without the overlying cortical plate (anatomical noise). The reconstructed slices are geometrically accurate. The periapical lesions do not change size or disappear on reconstructed scans as can happen with IOPA radiography. CBCT gains an unabridged view of dental anatomy, thus eliminating some of the most prevalent problems, such as superimposition and distortion. CBCT reduces false diagnosis and is rapidly replacing other radiographic techniques in diagnosis, quality control of treatment methods and techniques, and outcome assessment. 

A few studies have shown that CBCT imaging is not a reliable diagnostic method for differentiating radicular cysts from granulomas while histopathological evaluation by surgical biopsy remains the standard procedure [12]. The CBCT scan differentiates only a solid soft tissue lesion from one that has soft tissue plus an area that is less dense, that is, cavity with fluid, semisolid substance in the lumen. However, one study revealed that CBCT may be clinically more accurate and more useful than the biopsy because of the shortcomings in histological technique. Other studies have concluded that the detection of periapical lesions was considerably higher with CBCT than with IOPA radiography. Not only can the presence of a periapical lesion be diagnosed with CBCT, but the specific root that it is associated with can also be confirmed, which influences treatment planning. Thus, CBCT was found to be an accurate diagnostic method to identify large periapical lesions based on the gray scale value and provide essential diagnostic information for the management of this complex endodontic problem as compared to IOPA radiographs [1, 8]. To get an accurate reading, the whole lucency should be scanned for the most lucent or least dense area. Negative grayscale value indicates lumen of a bay or true cyst (cavitated lesions). Positive grayscale value indicates epithelioid granuloma or a granuloma. However, it is not clear whether all CBCT scan-detected periapical lesions are true lesions [3]. 

Recently, limited field of view CBCT has proved to be useful in detecting periapical lesions in the maxillary region in a case report. The knowledge of the relationship of periapical lesions to the roots of maxillary teeth may be useful in treatment planning and in the prevention of complications that may occur during apical surgery. A study revealed that 34% of the lesions detected with CBCT were missed with IOPA radiography in maxillary premolars and molars [6].

It is essential to understand which factors positively or negatively influence the outcome of endodontic treatment. IOPA radiographs were used in most previous outcome studies. However, periapical lesions could be radiographically undetectable when lesions are confined within the cancellous bone and covered by a thick cortex. A study compared the endodontic outcome predictors identified with IOPA radiography and CBCT revealed that CBCT was more sensitive than IOPA radiography in detecting extra canals and posttreatment periapical lesions [7]. The information obtained by CBCT evaluation of periapical repair following root canal treatment was comparable to histological analysis, whereas IOPA radiographs underestimated the size of the periapical lesion [2]. Absence of radiolucency in IOPA radiograph does not guarantee a healthy periapex. CBCT scan is better suited for detection and diagnosis of periapical lesions. CBCT is a technology that has proven useful for localization and characterization of root canals and treatment planning of periapical surgery. This case report showed that CBCT is a good noninvasive modality towards the differential diagnosis of periapical lesions. 

In both cases, the large lesions were treated by endodontic surgery (apicoectomy). Although CBCT revealed the lesion in the first case to be a periapical granuloma, due to its large size in all the three dimensions, it was decided to treat it surgically. The rationale was that according to the literature, endodontic surgery done for large lesions has a more favourable and predictable treatment outcome, and the periapical healing is faster, compared to root canal treatment alone. Moreover, the true size, extent, and location of the lesion obtained by CBCT help in the treatment planning for surgery and make it a safe procedure.

Following endodontic treatment and surgery of a periapical lesion, to be able to determine if the procedure is successful or not, healing of lesions is followed up by radiographic imaging. This can be done by observing changes in the periapical radiolucencies. Healing assessment using conventional and newer three-dimensional imaging includes, but is not limited to, periapical osseous lesions, status after endodontic surgery, and hard tissue deposition in regeneration procedures. Due to a low predictive value of periapical radiographs to distinguish between periapical disease and health, future assessment of endodontic treatment efficacy may include 3D imaging from small field-of-view CBCT units [14]. In a study, the diagnosis using CBCT revealed a lower healed and healing rate for root canal treatment than IOPA radiographs, particularly in roots of molars at the end of one year [15].

The use of CBCT is advocated in cases where the benefits of this investigation outweigh any potential risks to the patient, such as radiation exposure. Another limitation of CBCT is that an endodontic expert is required to analyze the whole lesion manually to search for cystic cavity; thus it is time consuming and prone to human error. Future improvements in CBCT technology may include systems with even more favorable diagnostic yields, lower radiation exposure, and reduced price. For now, CBCT imaging can be seen as a highly useful and, in some situations, an indispensable part of the modern dental imaging armamentarium. 

## 4. Conclusion

This case report revealed that CBCT scan may provide a better, more accurate, and faster method to differentially diagnose a solid from a fluid-filled large periapical lesion or cavity as compared to conventional IOPA radiographs. CBCT also influences the treatment planning and management of such large lesions and is useful for long-term assessment of the outcome of endodontic surgery.
